# Effects of Knee Joint Angle and Contraction Intensity on the Triceps Surae Stiffness

**DOI:** 10.3389/fbioe.2022.913423

**Published:** 2022-06-22

**Authors:** Ming Lin, Weixin Deng, Hongying Liang, Suiqing Yu, Qin Xu, Chunlong Liu

**Affiliations:** Clinical Medical College of Acupuncture, Moxibustion and Rehabilitation, Guangzhou University of Chinese Medicine, Guangzhou, China

**Keywords:** knee joint angle, the triceps surae, active stiffness, shear wave elastography, biomechanical analysis

## Abstract

**Purpose:** Monitoring the contractility of muscles assists the clinician in understanding how muscle functions as part of the kinetic system. This study investigated the effect of knee joint angles under different resistance on the stiffness of the medial gastrocnemius (MG), lateral gastrocnemius (LG), and soleus (SOL) muscles using the shear wave elastography (SWE) technique.

**Methods:** A total of 22 females were recruited. During isometric plantar flexion, at knee 0-degree (fully extended) and knee 90-degree (flexed 90°), the shear modulus on the MG, LG, and SOL was measured by shear wave elastography at no contraction and two intensities (40% and 80%) of maximal voluntary contraction (MVC). Shear modulus is a mechanical parameter to describe stiffness, and stiffness is a proxy for muscle contractility.

**Results:** There were moderate-to high-positive correlations between the active stiffness of triceps surae muscles and isometric contraction intensity (r: 0.57–0.91, *p*＜0.001). The active stiffness in MG and LG with extended knees was higher than that with flexed knees (*p*＜0.001). The active stiffness in SOL with flexed knee was higher than that with extended knee (*p*＜0.001).

**Conclusion:** Active stiffness can be considered a quantitative indicator generated by the force output of the triceps surae. Different knee joint angles cause three triceps surae muscles to exhibit non-uniform mechanical properties, which may explain part of the mechanism of soft tissue injury during physical exercise.

## Introduction

The triceps surae are the important muscles for humans to be able to perform upright movements. Injury, performance, and training of the triceps surae have been a popular issue in current sports and sports science research (Peake et al., 2017; Liu et al., 2020). After the Achilles tendon rupture, the lack of strength in the triceps surae is one of the signs of impaired ankle function (Sun et al., 2020). Forces of passive muscle tension and active muscle contraction act directly on the skeleton during human movement (Akuzawa et al., 2017). Control of motions is adjusted by the capacity of muscle contraction. Stability and flexibility of the lower extremity joints are also affected by its musculoskeletal system (Ham et al., 2020). The contraction capacity of muscle determines the movement pattern and the strategy which is adopted by the body in response to various conditions (Bach et al., 1983). Therefore, monitoring the contraction capacity of the triceps surae assists the clinician in understanding how muscle functions as part of the kinetic system.

The voluntary contraction of the triceps surae creates the bulk of the plantar flexion torque in the mid–late gait stages (Li et al., 2018). Isometric plantar flexion can maximize activity of the triceps surae. The triceps surae comprises the medial gastrocnemius (MG), lateral gastrocnemius (LG), and soleus (SOL). The differences in fiber types and anatomical structures result in different mechanical properties of three triceps surae muscles during plantar flexion. The SOL has a higher percentage of slow muscle fibers (70%), while both the MG and LG contain approximately 50% of slow muscle fibers (Edgerton et al., 1975), indicating that the contractility of the gastrocnemius is stronger than that of the SOL. In addition, the MG and LG insert above the knee joint, whereas the SOL inserts below the knee. Changes in knee angles appeared to affect the determinants of three triceps surae muscle behavior, including slack length of the series elastic element, mean moment arm, maximum force, and length of the contractile element (Out et al., 1996; Akasaka et al., 2004). In general clinical palpation, the higher muscle contraction intensity is, the stiffer muscle is. In the case of the triceps surae during varied locomotion tasks, it can be speculated that contraction capacity of muscle readjustment contributes to optimized function by enabling for more exact exertion of joint moments from the several muscle actuators that span the ankle and subtalar joints. In clinical practice, a dynamometer is a kind of popular quantifiable appliance for sports and rehabilitation training as it is simple to use, portable, and cost-effective. However, it can only be used to measure the strength of certain synergistic muscle groups (Ashall et al., 2021; Tanveer et al., 2021), and it cannot be utilized to establish biomechanical studies on individual muscles *in vivo*.

Recently, as a non-invasive and radiation-free imaging method, shear wave elastography (SWE) has been widely used to assess the relationship between local muscle stiffness and contractility. The shear modulus and normalized joint torque with isokinetic dynamometry showed a good linear relationship in the rotator cuff muscle (Kim et al., 2018). Previous research has discovered that muscle stiffness is proportional to contractility (Mendes et al., 2018; Wang L. et al., 2020). SWE is based on Hooke’s law, and muscle stiffness is described as the slope between changes in force and muscle deformation (Creze et al., 2018). SWE uses shear wave velocity to quantify local muscle stiffness: *μ* = 3ρ·v^2^ (*μ*: shear modulus, ρ: tissue density, and v: shear wave velocity). Shear modulus is a mechanical parameter to describe stiffness, and stiffness is a proxy for muscle contractility. Our previous study shows that SWE is a reliable method for measuring the triceps surae stiffness (Zhou et al., 2019). To the authors’ knowledge, stiffness redistribution produced by muscular contraction at different knee angles has not been proven. Muscle stiffness redistribution can reflect muscle force output under neurological modulation, which helps clinicians to monitor muscle contractile performance and design more efficient physical training programs for patients.

It can be hypothesized that changing the knee joint angle can alter the active stiffness of three triceps surae muscles, allowing the body to be more economical and accurate in responding to different functional tasks. Thus, the aim of this study was to assess the active stiffness of the MG, LG, and SOL at different knee postures during isometric plantar flexion.

## Materials and Methods

### Ethics Statement

All procedures were approved by the Ethics Committee of Guangdong Provincial Hospital of Traditional Chinese Medicine (YE 2020-329-01) and carried out following the guidelines of the Declaration of Helsinki. All recruited subjects were informed of the experimental procedures and the safety of SWE in the present study. Each subject signed an informed consent form.

### Subjects

A total of 22 healthy female college students (mean age: 20.4 ± 0.8 years old, height: 160.05 ± 2.47 cm, and weight: 51.20 ± 4.04 kg) were recruited. The dominant leg was defined as the leg used more for kicking. Only triceps surae muscles of the right calf were measured. All subjects were identified as right leg–dominant. Because the instrumentation has a small threshold for musculoskeletal patterns that make it difficult to monitor larger muscle stiffness in male subjects, only female subjects were recruited for this study. Inclusion criteria were as follows: all subjects were healthy and able to follow the operator’s instructions. Exclusion criteria were as follows: anybody with a lower limb neuroskeletal muscle injury, such as Achilles tendon injury, heel discomfort, plantar fasciitis, or an anterior cruciate ligament (ACL) injury, and individuals taking corticosteroid medication.

### Tools and Equipment

All ultrasound examinations were performed by an ultrasound SWE system (Aixplorer Supersonic Forigin, France) with a 50-mm linear array transducer (SL15-4, Supersonic Forigin, France), using the instrument’s default standard musculoskeletal (MSK) settings. Other settings of the SWE mode were as follows: penetration mode with 85% opacity, and the middle part of the muscle thickness was chosen for measurement. The size of the region of interest (ROI) was set to 10 × 10 mm. The Q-box diameter was set to 5 mm. Change from blue (soft) to red (hard) was based on shear modulus. The measurable range was adjusted to 0–300 kPa.

A micro FET2 hand-held dynamometer (HHD) (Hogan Science, Salt Lake City, UT, United States) was used to measure force during plantar flexor isometric contraction. The HHD was anchored on the wall as in the previous study (Krasnow et al., 2011), using a hook and loop fastener for height adjustability and stability. The HHD pad was placed on the first metatarsal, which transforms the pressure data into lbs through the pressure sensor and displays it on the screen.

### Measurement Positions

**FIGURE 1:**
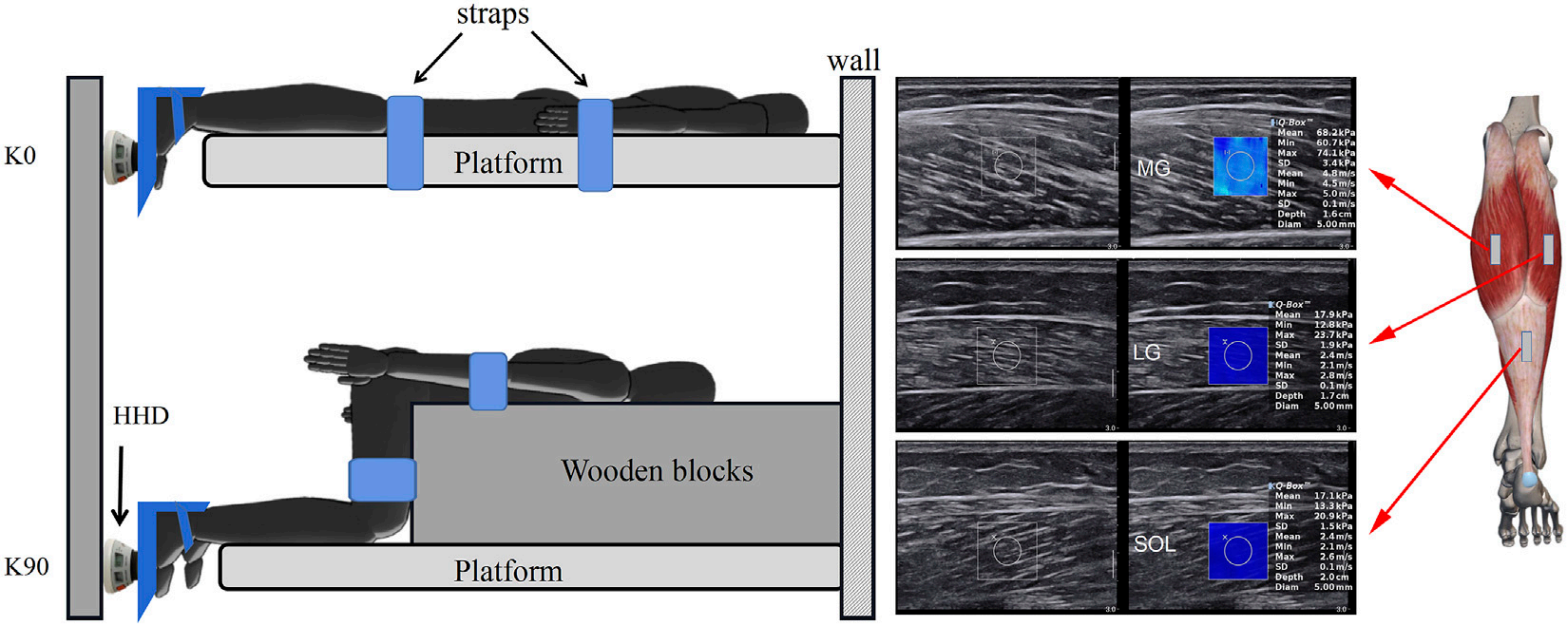
Schematic diagram of the shear modulus of triceps surae components during isometric plantar flexion. In the extended knee (K0), a subject was prone and the trunk was secured to the platform using straps. In the flexed knee (K90), a heavy wooden block was placed in front of the thigh to avoid displacement of the lower leg during isometric contraction. The force of the ankle plantar flexors was measured at 0° (neutral position) using a hand-held dynamometer (HHD) that was placed on the first metatarsal. MG: medial gastrocnemius; LG: lateral gastrocnemius; and SOL: soleus.

In the 0-degree knee (K0), the trunk was secured by straps on the platform, with the hip and knee joints in full extension; in the 90-degree knee (K90), a wooden block was placed in front of the thigh, with the hip and knee joints at 90°. The trunk and thighs were secured by straps to a wooden block (with loop hooks) to prevent body movement. The regions of the MG, LG, and SOL were identified by the ultrasound B-mode. The MG and LG were at 30% (proximal) of calf-length (the MG’s length is measured from the popliteal fossa to the lateral malleolus), where the cross-sectional areas of the gastrocnemius are nearly maximum. The length of the LG is measured from the popliteal fossa to the medial malleolus. A tape was used to measure the length, and a black pen was utilized to mark the location of the measurement site (Zhou et al., 2019)). The SOL at 70% (distal SOL) of calf-length (Akiyama et al., 2016) (Figure 1) marked the skin in these positions with a waterproof marker. The sequence of imaging was MG, LG, and SOL. The ankle joint was stabled at a neutral position with a 0° in plantar flexion.

### Procedures

In the pilot test, the maximal voluntary contraction (MVC) force of the plantar flexors was measured at neutral ankle position using a HHD (Figure 1). In addition, two repeated attempts for each knee posture and 1 minute of rest was allowed between two attempts. The mean value of two attempts was recorded as the maximal intensity of isometric plantar flexion. One MVC was recorded for each knee posture. Moreover, 3 min of rest between two knee postures was allowed. The posture of the knee joint was randomized. Visual feedback and strong oral encouragement were given for all attempts. Subjects were instructed to contract only the plantar flexors. Based on the isometric plantar flexion force generated during the MVC (100%), 40% and 80% of the MVC were viewed as low and high loads in respect to functional tasks such as walking and hopping (Kelly et al., 2018).

Measurement of the muscle stiffness at no contraction and two intensities of MVC after 30 min of the pilot test. 1) Without contraction: the degree of the muscle stiffness was measured with a position of the knee extension or knee flexion. To reduce experimental errors, subjects were explicitly asked to keep the lower extremity fully relaxed (Liu et al., 2020). 2) Isometric contraction: an assistant instructed the subject to perform isometric plantar flexion. Subjects did not view the %MVC, and they were instructed to hold at a particular level of MVC by the assistant. While the target (40% or 80% of MVC) force was displayed on the HHD, the examiner measured the stiffness of the muscle. The probe was placed on one muscle, and then the experiment was repeated with the probe placed on the other muscle. The probe was gently placed on top of a large amount of coupling agent at the marker point. The targeted muscle fibers were parallel. The sequence of the procedure was without contraction, 40% contraction, and 80% contraction. Subjects were required to maintain a target force for the duration of at least 5 s. Then, the subjects were asked to relax for 1 min between each measurement to avoid muscle fatigue. A sub-threshold range of ±2.5%MVC was set up for the 40% and 80% targets to account for the observed variation in contraction control (Fukutani et al., 2012). Three values of shear modulus were recorded for each measurement. The average values were used for further analysis.

### Statistical Analysis

A sample size of 4 (22 subjects included) was deemed sufficient to achieve high statistical power. The effect sizes for MVC were determined from our pretest (eight subjects). A effect size of 3.69 was calculated by the sample size calculator G∗Power 3.1.9.2, alongside a power of 95% and a significance level of 0.05. The experience of our previous study has also been taken into account (n = 20; Chen et al., 2022).

SPSS 18.0 (SPSS Inc., Chicago, IL, United States) was used for statistical analysis of all the data. The Shapiro–Wilk test was used to check the normal distribution of all stiffness data. All stiffness data were expressed as mean ± standard deviation. All stiffness values were normally distributed. A 2-way (2 knee positions × 3 contraction intensities) analysis of variance (ANOVA) was performed to evaluate the data to discover if differences exist in muscle stiffness between the extended and flexed knee when comparing various contraction intensities. If there was a knee position × contraction intensity interaction, the main effects were investigated and a Bonferroni correction was applied on the post hoc tests. The plantar flexion force was divided into two stages: the low-load stage (no contraction (0%)–40%) and the high-load stage (40%–80%). The validity of shear modulus was evaluated using general linear model analysis. The slope of the stiffness is the regression coefficient of the general linear equation, which indicates the level of change in stiffness when altering the contraction intensity. Correlation between muscle stiffness and plantar flexion intensity was assessed using Pearson’s correlation analysis. The correlation degree of the correlation coefficient (r-value) was set to |r| > 0.8 for high correlation, 0.5 <|r| ≤ 0.8 for moderate correlation, 0.3 <|r| ≤ 0.5 for low correlation, and |r| ≤ 0.3 for no linear correlation. Paired t-tests were applied to verify differences in muscle stiffness between knee extension and flexion. Cohen’s d was used to examine the effect size. The significance level of the statistical data difference was set as *p* = 0.05.

## Results

### Shear Modulus of Three Triceps Surae Muscles Between Extended and Flexed Knees

**FIGURE 2:**
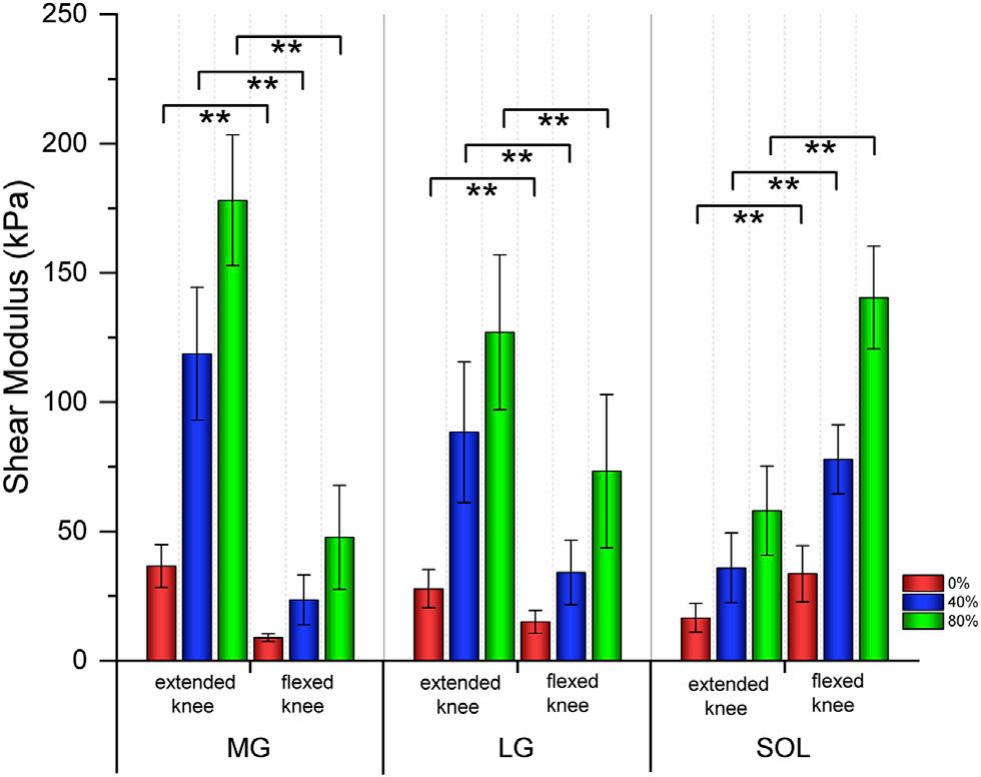
Bar graphs of the shear modulus of three triceps surae muscles between extended and flexed knee. ** means significant intergroup difference (*p* < 0.001). Red, blue, and green represent no contraction (0%), 40%, and 80% maximum voluntary contraction (MVC) during isometric plantar flexion, respectively. MG: medial gastrocnemius; LG: lateral gastrocnemius; and SOL: soleus.

Table 1 shows the relationship in the shear modulus of the MG, LG, and SOL between extended knees and flexed knees. As the intensity of isometric plantar flexion increased, the stiffness of the MG, LG and SOL became harder. Both the MG and LG with extended knees were stiffer than those of flexed knees (*p*＜0.001); the SOL with a flexed knee was stiffer than that of an extended knee (*p*＜0.001) (Figure 2).

**TABLE 1:** Shear modulus of the triceps surae muscle with different knee postures in different isometric contraction (plantar flexion) intensity.

Muscles	Contraction intensity	K0 (kPa)	K90 (kPa)	*p*	Cohen’s d
MG	Without contraction	36.58 ± 8.26	8.83 ± 1.50	<0.01	4.67
	40% of MVC	118.70 ± 25.70	23.45 ± 9.62	<0.01	4.91
	80% of MVC	178.10 ± 25.32	47.66 ± 20.11	<0.01	5.70
LG	Without contraction	27.80 ± 7.37	14.97 ± 4.40	<0.01	2.11
	40% of MVC	88.34 ± 27.19	34.08 ± 12.44	<0.01	2.56
	80% of MVC	127.02 ± 29.94	73.30 ± 29.65	<0.01	1.80
SOL	Without contraction	16.48 ± 5.56	33.60 ± 10.89	<0.01	−1.98
	40% of MVC	35.87 ± 13.48	77.84 ± 13.34	<0.01	−3.12
	80% of MVC	57.98 ± 17.30	140.43 ± 19.81	<0.01	−4.43

K0: Knee 0°, K90: Knee 90°, MG: medial gastrocnemius, LG: lateral gastrocnemius, SOL: soleus.

### Variations in the Shear Modulus of Muscles With Different Levels of Plantar Flexion

**FIGURE 3:**
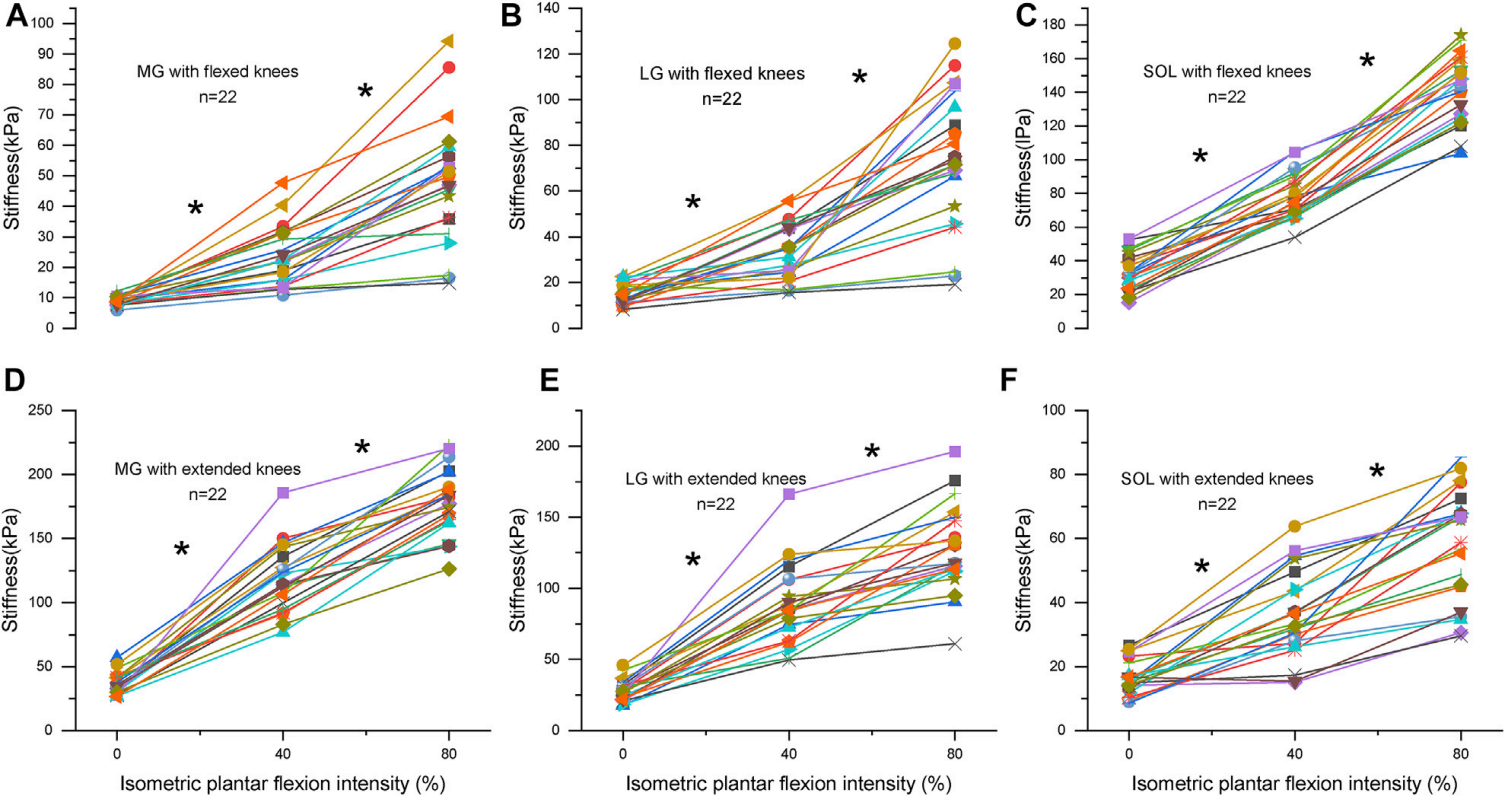
**(A–C)** represent the stiffness of the medial gastrocnemius (MG), lateral gastrocnemius (LG) and soleus (SOL) with flexed knees, respectively. **(D–F)** represent the stiffness of the MG, LG and SOL with extended knees, respectively. Changes in stiffness is related to isometric contraction intensity (0%: no contraction, 40%, and 80% of maximum voluntary isometric contraction) for triceps surae muscles in 22 subjects. Each subject is represented by a different symbol. * Significant (*p* < 0.05) at different contraction intensities.

There is a knee position × contraction intensity interaction, the main effects are investigated, and a Bonferroni correction is applied on the post hoc tests in Supplementary Table S1. Table 2 shows the relationship between the stiffness of triceps surae muscles and the intensity of isometric plantar flexion. There were moderate-to-high positive correlations between the stiffness of triceps surae muscles and isometric plantar flexion intensity (r: 0.57–0.91, *p*＜0.001). At flexed knees, the slope of the MG, LG, and SOL at the high-load stage increased by 65%, 105%, and 41% compared to that of the low load stage, respectively. But the increase of the MG and LG in extended knees was blunted during the high-load stage, and the slope of the MG and LG at the high-load stage decreased by 27% and 36% compared to that of the low load stage, respectively. Figure 3 shows the relationship between the intensity and stiffness of the plantar flexion in the MG, LG, and SOL with knee flexion and extension, respectively, in all subjects (n = 22).

**TABLE 2:** Correlation between muscle stiffness and isometric contraction intensity.

Muscles	Knee	Low-load stage			High-load stage		
		Slope (kPa/%MVC)	R^∧^^2	Pearson’s r	Slope (kPa/%MVC)	R^∧^^2	Pearson’s r
MG	K0	2.05 ± 0.64	0.83	0.91*	1.48 ± 0.59	0.58	0.76*
	K90	0.36 ± 0.23	0.54	0.73*	0.61 ± 0.36	0.38	0.62*
LG	K0	1.51 ± 0.63	0.71	0.84*	0.97 ± 0.59	0.32	0.57*
	K90	0.47 ± 0.31	0.52	0.72*	0.98 ± 0.63	0.44	0.66*
SOL	K0	0.48 ± 0.30	0.48	0.69*	0.55 ± 0.31	0.34	0.59*
	K90	1.11 ± 0.31	0.77	0.88*	1.56 ± 0.44	0.78	0.88*

K0: knee 0°, K90: knee 90°, MG: medial gastrocnemius, LG: lateral gastrocnemius, SOL: soleus, Low-load stage: without contraction ∼40% of MVC, High-load stage: 40%–80% of MVC, *p＜0.05.

## Discussion

The main findings of our study were that 1) he slope of the MG stiffness with an extended knee was larger than that with a flexed knee, and the slope of the SOL stiffness with flexed knees was larger than that with extended knees. 2) The stiffness of the MG and LG with an extended knee was larger than that with a flexed knee. The stiffness of the SOL in a flexed knee was larger than that in an extended knee. 3) There was a moderate-to-high positive linear relationship between the stiffness of muscle and the intensity of isometric plantar flexion.

During the high-load stage, the slope of MG stiffness with flexed knees was larger than that with extended knees. The slope of SOL stiffness changes with an extended knee was larger than that with a flexed knee. This was the first trial to compare the active stiffness recruitment of three triceps surae muscles at different knee postures. First, the pennation angle between the fiber and fascia of the MG is ∼60° (Hirata et al., 2016). According to the trigonometric function relationship, the force is transmitted to the tendon by the cosine torque of the pennation angle. The factor for the MG is 0.5. In other words, only half of the force is transmitted to the Achilles tendon. In addition, muscles operate with high gear ratios during the low-load stage; fibers rotate to larger angles of pennation, which consume part of the force. Muscles operate with lower gear ratios during the high-load stage; fibers rotate lightly, helping conserve force (Bach et al., 1983). Thus, the contribution of the MG with extended knees at the high-load stage would be smaller than that at the low-load stage. Second, the possibility of neural mechanisms interfering with the SOL cannot be ruled out (Lauber et al., 2014). During isometric plantar flexion with flexed knees, the contractility of the gastrocnemius muscle is diminished. The recruitment increased in the SOL can compensate for the loss of recruitment produced by the MG. From a physical standpoint, softer or stiffer materials exhibit greater or less compliance (Ditroilo et al., 2011). Softer gastrocnemius with flexed knees indicates higher compliance, allowing knees to be more flexible in extension. Knee hyperextension is avoided by strengthening the stiffness of gastrocnemius with extended knees. However, if the stiffness of the gastrocnemius is insufficient, shear loads are distributed to the anterior cruciate ligament (Morgan et al., 2014), leading to ligament injury during sports. This would suggest that optimum stiffness of the gastrocnemius is essential for knee stability and flexibility.

Although both the MG and LG have 50% of slow muscle fibers (Edgerton et al., 1975), we also found that the slope in the MG stiffness with an extended knee was larger than that in the LG. The result could be described by their architectural properties. First, the physiological cross-sectional area in MG is 2.1 times more than that in LG (Albracht et al., 2008). Second, the Achilles tendon consists of tendons originating from the SOL, MG, and LG. According to Pękala et al. (2017), the LG and MG tendon twist 135.98 ± 33.58° and 28.17 ± 15.15°, respectively. Tendons with higher torsional angles sacrifice more elastic energy during plantar flexion, resulting in less force transferred to the LG and less stiffness recruited from the LG.

We also compared the relaxed stiffness of the SOL at two knee angles. Contrary to our previous study results in male subjects (Liu et al., 2020), the relaxed stiffness of the SOL at flexed knees was higher than that at extended knees for female subjects in the current study. One study showed that males have more septal connections between the superficial fascia and the dermis than females (Rudolph et al., 2019). This suggests that males are more susceptible to fascial force transmission. Males show a tighter connection between connective tissue and muscle bellies during passive extension of the knee joint. The distal SOL was pulled by upward stress from the gastrocnemius fascia and resulted in a tighter SOL. The increased tension in the distal SOL makes itself stiffer; thus, the SOL with extended knees exhibited higher stiffness in males.

Muscle contraction is powered by the interaction capacity of myosin molecules with actin filaments (Kaya and Higuchi et al., 2013), which in turn alters the active stiffness in macro conditions. SWE quantifies and visualizes the distribution of active stiffness caused by muscular contraction. Our results showed that there were moderate-to-high positive correlations between muscle stiffness and plantar flexion intensity. It has been demonstrated that the stiffness in the rectus femoris increases with the increase of impedance (*p* < 0.001) (Tang et al., 2020). At an isometric trunk extension task, Murillo et al. observed that multiple muscle stiffness increased as resistance load increased (Murillo et al., 2019). These studies showed that changes in active stiffness could be described as an alternative indicator of the degree of muscle contractility.

Microscopic-level analyses of the active stiffness production indicate that it occurs in an environment of tissues that exhibit spring-like behavior. Muscle myosins are highly efficient motors that adjust the power output according to loads. When the load increases, it elicits a stronger binding between myosin and actin (termed the “cross-bridge”) (Cheng et al., 2020) and decreases the detachment rate of myosin molecules (Veigel et al., 2005). Also, the active stiffness is regarded as mechanical information for the force driven by cross-bridges (Wang T. et al., 2020). Excessive or insufficient stiffness might provide the wrong information to the nervous system, which would result in uncoordinated muscle contractions and an increased risk of soft tissue injuries. The Achilles tendon is the conjoined tendon of the SOL, LG, and MG. They are identified as discrete components during anatomical dissection. There are different stiffness recruitment of three triceps surae muscles in different knee postures. The MG showed larger stiffness with extended knees in our result. Moreover, the smallest component of the Achilles tendon is the sub-tendon from the MG (27.68%) (Pękala et al., 2017). These factors suggest that the sub-tendon from the MG is more vulnerable to injury due to its tighter movements, which may explain how the tear of a single sub-tendon occurs on the stage from flexed to extended knees (Thermann, 2019).

We also considered that a lower coefficient of fitting (R-squared range: 0.32–0.78) appeared at the high-load stage in the current research. Our results agreed with a study by Inami et al. who found that the force–stiffness relationship was not linear (Inami et al., 2017). However, Ateş et al. (2015) indicated a high coefficient of fitting (R-squared range: 0.86–0.98) of the relationship between stiffness and Abductor Digiti Minimi torque. A high R-squared indicates the fit of the regression model is excellent. There are several explanations for this discrepancy. First, this variation can be justified by the deformation of foot soft tissue during a higher level of plantar flexion. Inevitable ankle joint angular rotation affects muscle torque (Karamanidis et al., 2005). The study by Hessel et al. (2021) illustrated that changes in torque and force moment may have a potential effect on muscle contractility. The muscle output strategy is altered to maximize performance on the various task demands. Second, the possible interference of force transmission between sub-tendons cannot be ruled out (Gains et al., 2020). Therefore, the active stiffness recruitment might be adjusted to make the output power more economical at a specific knee position.

SWE provides a quantitative assessment of mechanically driven muscle recruitment in macroscopic conditions. Active stiffness can be regarded as a parameter indicator of muscle contraction capacity for physiotherapy or therapeutic feedback in the fields of sports science and rehabilitative training. SWE provides additional information on voluntary muscle contraction by adding stiffness as another measurable characteristic of current ultrasound imaging techniques. As far as we are concerned, the exact proportion of fibers (Liu et al., 2019) and muscle–tendon interaction force could not be determined *in vivo*. Further studies will investigate other factors that affect stiffness recruitment.

## Limitations

There were several limitations in the present study. First, the mechanical behavior of the Achilles tendon may also influence the active stiffness of the triceps surae. The stiffness of the Achilles tendon exceeded the threshold of the SWE’s device. We were unable to explore the biomechanical link between the triceps surae and the Achilles tendon *in vivo*. Second, although we instructed the subjects not to contract auxiliary muscles (e.g., quadriceps), we were unable to confirm that this was the case due to the lack of electromyography (EMG) equipment. In the next trial, we will introduce an EMG device to objectively monitor muscle activity. Finally, in our preliminary experiments, muscle stiffness in male subjects with 80% MVC often exceeded the measurement range of the instrument. Therefore, only female individuals were recruited in this study. Finally, the results of this study apply only to healthy young people. In the future, we will progressively conduct studies on injured patients.

## Conclusion

In summary, SWE can be used to quantify active stiffness changes in the triceps surae during muscle contraction. Knee joint angles can affect the stiffness recruitment of the MG, LG, and SOL. The MG and LG with extended knees showed higher stiffness at the low-load stage, whereas the SOL with flexed knees exhibited higher stiffness at the high-load stage. Non-uniform stiffness increase in MG, LG, and SOL may explain part of the mechanism of soft tissue injury. Further studies are necessary to evaluate the stiffness recruitment of other muscles in different functional tasks.

## Data Availability

The original contributions presented in the study are included in the article/Supplementary Material; further inquiries can be directed to the corresponding authors.
