# Supplementary material for: Effects of Knee Joint Angle and Contraction Intensity on the Triceps Surae Stiffness
Source: Front Bioeng Biotechnol. 2022 Jun 22;10:913423. doi: 10.3389/fbioe.2022.913423 (PMC9256962; doi:10.3389/fbioe.2022.913423)
Supplement: Supplementary file 1 [file Table1.DOCX]

| Supplementary Table 1. Main effects test for the triceps surae stiffness | | | | | | |
| --- | --- | --- | --- | --- | --- | --- |
|  | | Sum of Squares | Degrees of Freedom | Mean Square | F | p |
| MG | Knee Angle | 235507.1489 | 1 | 235507.1489 | 755.6403 | 0.000 |
|  | MVC | 179216.2718 | 2 | 89608.1359 | 287.5136 | 0.000 |
|  | Knee Angle*MVC | 59917.8379 | 2 | 29958.9189 | 96.1251 | 0.000 |
| LG | Knee Angle | 53520.9778 | 1 | 53520.9778 | 117.0204 | 0.000 |
|  | MVC | 136521.0743 | 2 | 68260.5371 | 149.2476 | 0.000 |
|  | Knee Angle*MVC | 12421.6852 | 2 | 6210.8426 | 13.5796 | 0.000 |
| SOL | Knee Angle | 73457.3728 | 1 | 73457.3728 | 366.788 | 0.000 |
|  | MVC | 121824.2089 | 2 | 60912.1044 | 304.1469 | 0.000 |
|  | Knee Angle*MVC | 23911.6220 | 2 | 11955.8110 | 59.6978 | 0.000 |
