# Supplementary material for: Effects of Knee Joint Angle and Contraction Intensity on the Triceps Surae Stiffness
Source: Front Bioeng Biotechnol. 2022 Jun 22;10:913423. doi: 10.3389/fbioe.2022.913423 (PMC9256962; doi:10.3389/fbioe.2022.913423)
Supplement: Supplementary file 2 [file Table2.DOCX]

| Supplementary Table 2. Post-hoc comparisons results for the triceps surae stiffness | | | | | |
| --- | --- | --- | --- | --- | --- |
|  | (I) MVC | (J) MVC | Mean Difference(I-J) | Std. Error | p |
| MG | 0%MVC | 40%MVC | -48.3735 | 3.76386 | 0.000 |
|  |  | 80%MVC | -90.1765 | 3.76386 | 0.000 |
|  | 40%MVC | 80%MVC | -41.8030 | 3.76386 | 0.000 |
| LG | 0%MVC | 40%MVC | -39.8226 | 4.55953 | 0.000 |
|  |  | 80%MVC | -78.7734 | 4.55953 | 0.000 |
|  | 40%MVC | 80%MVC | -38.9507 | 4.55953 | 0.000 |
| SOL | 0%MVC | 40%MVC | -31.8151 | 3.01716 | 0.000 |
|  |  | 80%MVC | -74.1651 | 3.01716 | 0.000 |
|  | 40%MVC | 80%MVC | -42.3500 | 3.01716 | 0.000 |
